# Health related quality of life outcomes for unresectable stage III or IV melanoma patients receiving ipilimumab treatment

**DOI:** 10.1186/1477-7525-10-66

**Published:** 2012-06-13

**Authors:** Dennis A Revicki, Alfons JM van den Eertwegh, Paul Lorigan, Celeste Lebbe, Gerald Linette, Christian H Ottensmeier, Shima Safikhani, Marianne Messina, Axel Hoos, Samuel Wagner, Srividya Kotapati

**Affiliations:** 1 United BioSource Corporation, 7101 Wisconsin Avenue, Suite 600, Bethesda, MD, 20814, USA; 2 VU University Medical Center, Boelelaan 1117 1081HV, Amsterdam, The Netherlands; 3 University of Manchester, Christie NHS Foundation Trust Wilmslow Road, Manchester, M20 4BX, UK; 4 Hôpital St. Louis, APHP Dermatology University Paris 7, Diderot, France; 5 Division of Oncology, Washington University School of Medicine, 660 S, Euclid Avenue, Campus Box 8056, St. Louis, MO, 63110, USA; 6 Southampton University and University Hospital Southampton, Cancer Sciences Division, Southampton, O16 6YD, UK; 7 Bristol-Myers Squibb, 5 Research Parkway, Wallingford, CT, 06492, USA; 8 Bristol-Myers Squibb, 100 Nassau Park Boulevard, Princeton, NJ08540, USA

**Keywords:** Ipilimumab, Randomized clinical trial, EORTC QLQ-C30, Advanced melanoma, Health-related quality of life

## Abstract

**Background:**

In an international, randomized Phase III trial ipilimumab demonstrated a significant overall survival benefit in previously treated advanced melanoma patients. This report summarizes health-related quality of life (HRQL) outcomes for ipilimumab with/without gp100 vaccine compared to gp100 alone during the clinical trial’s 12 week treatment induction period.

**Methods:**

The Phase III clinical trial (MDX010-20) was a double-blind, fixed dose study in 676 previously treated advanced unresectable stage III or IV melanoma patients. Patients were randomized 3:1:1 to receive either ipilimumab (3 mg/kg q3w x 4 doses) + gp100 (peptide vaccine; 1 mg q3w x 4 doses; ipilimumab plus gp100, n = 403); gp100 vaccine + placebo (gp100 alone, n = 136); or ipilimumab + placebo (ipilimumab alone, n = 137). The European Organization for Research and Treatment of Cancer Quality of Life Questionnaire (EORTC QLQ-C30) assessed HRQL. Baseline to Week 12 changes in EORTC QLQ-C30 function, global health status, and symptom scores were analyzed for ipilimumab with/without gp100 vaccine compared to gp100 alone. Mean change in scores were categorized “no change” (0–5), “a little” (5–10 points), “moderate” (10–20 points), and “very much” (>20).

**Results:**

In the ipilimumab plus gp100 and ipilimumab alone groups, mean changes from baseline to Week 12 generally indicated “no change” or “a little” impairment across EORTC QLQ-C30 global health status, function, and symptom subscales. Significant differences in constipation, favoring ipilimumab, were observed (p < 0.05). For ipilimumab alone arm, subscales with no or a little impairment were physical, emotional, cognitive, social function, global health, nausea, pain, dyspnea, constipation, and diarrhea subscales. For the gp100 alone group, the observed changes were moderate to large for global health, role function, fatigue, and for pain.

**Conclusions:**

Ipilimumab with/without gp100 vaccine does not have a significant negative HRQL impact during the treatment induction phase relative to gp100 alone in stage III or IV melanoma patients.

**Trial registration:**

Clinicaltrials.gov identification number NCT00094653

## Introduction

Advanced melanoma is a serious and life threatening cancer which has an impact on health-related quality of life (HRQL). According to the American Cancer Society, there were an estimated 68,130 new cases of melanoma and 8,700 deaths in the US in 2010, which accounts for almost three-fourths of all skin cancer deaths [1]. The median overall survival for patients with untreated advanced melanoma ranges between 6 to 9 months [1-6]. Cornish et al. recently demonstrated that the impact of melanoma on patient HRQL is comparable with other cancers [7].

Until the recent approvals for vemurafenib and ipilimumab, none of the currently approved treatments for advanced melanoma have shown overall survival benefit [3,8-18]. The focus of current treatment is on improving survival, managing symptoms, and improving HRQL outcomes [2,19]. Studies have shown that melanoma impacts psychological functioning (i.e., anxiety, depression, and vulnerability) [20-24]. In studies of advanced melanoma patients receiving treatment, melanoma patients also reported significant impairments in physical functioning and fatigue symptoms [20,25,26]. Treatment-related HRQL outcomes vary by HRQL instrument, study methods and design, study dropout rates, and disease progression rates. These factors need to be taken into consideration when interpreting the findings of HRQL studies in advanced melanoma.

Several clinical trials comparing treatments for advanced melanoma have included HRQL measures [14,20,26-36]. In general, these clinical studies demonstrate varied HRQL and symptom effects for different treatments, although the earliest studies demonstrate significant impairment to functioning [34,35]. However, most of these studies have considerable dropout rates and results are often restricted to the initial weeks of the clinical trial study. Dropouts are frequently observed in patients with significant toxicity or disease progression, and these missing data can make the follow-up HRQL outcomes appear better than they are in reality [37].

Ipilimumab is an anti-CTLA-4 monoclonal antibody with anti-tumor activity and has demonstrated statistically significant improvement in overall survival in a Phase III study (MDX010-20) in patients with previously treated unresectable stage III or IV melanoma [9]. Efficacy and safety data corresponding to the Phase II and III clinical trials in advanced melanoma have been reported elsewhere [9,12,38]. Overall, ipilimumab, alone or in combination with gp100, was tolerable in subjects with advanced metastatic melanoma with a generally manageable safety profile, which is consistent with safety demonstrated in previous studies of ipilimumab [9]. Study drug-related adverse events, regardless of etiology, were severe (≥ Grade 3) for 19.5%, 26.0%, and 12.1% of subjects treated with ipilimumab plus gp100, ipilimumab alone, and gp100 alone, respectively [9]. Immune-related adverse events were the most frequently reported drug-related adverse events [9]. The immune-related adverse events of ipilimumab are managed through administration of systemic glucocorticoids and other immunosuppressant agents along with adherence to treatment according to well established guidelines [39,40]. The majority of these immune-related adverse events occurred during the induction period of ipilimumab treatment. This report summarizes the HRQL outcomes during the 12 week treatment induction period of the ipilimumab Phase III clinical trial (MDX010-20). Assessment of the effects of ipilimumab in relation to overall HRQL is important and will allow oncologists to appropriately educate patients on the risks and benefits of treatment with this agent.

## Methods

### Study design

Study MDX010-20 was conducted in accordance with International Conference on Harmonisation-Good Clinical Practices and the Declaration of Helsinki. The study was approved by local regulatory authorities and institutional review boards and Ethics Committees at the participating sites, and all subjects provided written consent. The Phase III clinical trial (MDX010-20) was a double-blind, fixed dose study in 676 previously treated patients with advanced stage III or IV melanoma [9]. Patients in this trial were randomized 3:1:1 to receive either ipilimumab (3 mg/kg q3w x 4 doses) + gp100 (peptide vaccine; 1 mg q3w x 4 doses; ipilimumab plus gpl00, n = 403); gp100 vaccine + placebo (gp100 alone, n = 136); or ipilimumab + placebo (ipilimumab alone, n = 137). The main inclusion criteria were men and women aged ≥18 years, histological confirmed advanced stage III or IV melanoma, Eastern Cooperative Oncology Group (ECOG) performance status of 0 or 1, and life expectancy of at least four months. Key exclusion criteria included active symptomatic or asymptomatic untreated central nervous system (CNS) metastasis, primary ocular melanoma, or pregnant or breastfeeding women. Patients with stable, pre-treated CNS metastases were allowed in the study.

### Treatment regimen

Ipilimumab, at a dose of 3 mg per kilogram of body weight, was administered with or without gp100 every three weeks for up to four treatments (induction) [9]. In the gp100 groups, patients received two modified HLA-A* 0201-restricted peptides with incomplete Freund’s adjuvant (Montanide ISA-51): a gp100:209-217(210M) peptide, 1 mg injected in the right anterior thigh, and a gp100:280-288(288V) peptide, 1 mg injected in the left anterior thigh. These injections were given immediately after the intravenous infusion of ipilimumab or placebo.

Treatment was started on day 1 of Week 1, and additional treatment was received at Weeks 4, 7, and 10 if there was no intolerable toxicity, no rapidly progressive disease, and no significant decline in performance status. This included patients who developed new lesions and/or experienced growth in baseline lesions. Patients were offered additional courses of therapy (reinduction) if they had stable disease after Week 12 or a confirmed partial or complete response and no dose-limiting toxicity, or if they had disease progression with their assigned treatment regimen [9].

### Health-related quality of life measure

HRQL was evaluated using the European Organization for Research and Treatment of Cancer Quality of Life Questionnaire (EORTC QLQ-C30) [41,42]. The EORTC QLQ-C30 contains subscales for global health status, and physical, emotional, role, cognitive, and social function, with higher scores indicating better functioning [41,42]. Symptom subscales include pain, nausea/vomiting, fatigue, dyspnea, appetite loss, insomnia, diarrhea, and constipation (higher scores indicate greater symptom severity). Extensive evidence is available supporting the reliability, validity, and responsiveness of the EORTC QLQ-C30 in different cancer populations [42,43]. In the Phase III trial (MDX010-20), HRQL outcomes were self-administered at the clinical centers before any clinical procedures or physician interactions, including any discussion of imaging studies at baseline and Week 12.

### Statistical analyses

Baseline to Week 12 changes in EORTC QLQ-C30 function, global health status and symptom scores were calculated. Analysis of variance models were used to compare treatment differences for the HRQL outcomes. Since there is clinical interest in effectiveness and risks in older oncology patients, post hoc subgroup analysis of EORTC QLQ-C30 data by age (<65 years versus ≥65 years) was also conducted, and these analyses were compared to the results from the total sample (i.e., combined age group). Descriptive analyses are reported for the data, and no statistical tests were performed due to the ad hoc nature and relatively small sample sizes. Interpretations of the mean change in scores were categorized as “no change” (0–5 points), “a little” (5–10 points), “moderate” (10–20 points), and “very much” (>20 points), based on Osoba et al. [44]. When function and symptom scores showed either “no change” or “little change,” they were interpreted as reflecting no or minimal impact on patient HRQL [44].

## Results

### Demographic and clinical characteristics

Patients were randomly assigned to either the ipilimumab plus gp100 (n = 403), ipilimumab alone (n = 137), and gp100 alone (n = 136) treatment arms. Participants from all three arms (total n = 676) had a mean age of 56.2 ± 57.0 years and 59% were male (Table 1). The majority of participants had M1C stage at entry (n = 483; 71.4%) and almost all had an ECOG performance status score of 0 or 1 (n = 665; 98.4%). All of the participants had received prior treatment for advanced melanoma. Twelve percent of participants (n = 82; 12.1%) had CNS metastases at baseline.

**Table 1:** Baseline demographics and clinical characteristics

	**Phase III (MDX010-20)**			
	**Ipilimumab plus gp100**	**Ipilimumab Alone**	**gp100 Alone**	**Total**
	**(N = 403)**	**(N = 137)**	**(N = 136)**	**(N = 676)**
**Age (years), mean**	55.6	56.8	57.4	56.2
**Gender, n (%)**				
Male	247 (61)	81 (59)	73 (54)	401 (59)
**Melanoma stage, n (%)**				
M0	5 (1.2)	1 (0.7)	4 (2.9)	10 (1.5)
M1a	37 (9.2)	14 (10.2)	11 (8.1)	62 (9.2)
M1b	76 (18.9)	22 (16.1)	23 (16.9)	121 (17.9)
M1c	285 (70.7)	100 (73.0)	98 (72.1)	483 (71.4)
**Prior treatment for advanced melanoma, n (%)**	403 (100.0)	137 (100.0)	136 (100.0)	676 (100.0)
**ECOG PS, n (%)**				
0	233 (57.8)	72 (52.6)	70 (51.5)	375 (55.5)
1	165 (40.9)	64 (46.7)	61 (44.9)	290 (42.9)
2 or 3	5 (1.2)	1 (0.7)	4 (2.9)	10 (1.4)
Missing	0	0	1 (0.7)	1 (0.1)
**CNS metastases at baseline, n (%)**	46 (11.4)	15 (10.9)	21 (15.4)	82 (12.1)

### HRQL outcomes

In the Phase III study (MDX010-20), 95% had baseline HRQL assessments and Week 12 assessments were available for 236 (62%), 85 (65%), and 80 (61%) of the patients treated with ipilimumab plus gp100, ipilimumab alone, and gp100 alone, respectively. Missing HRQL data at baseline were due to administrative errors. Reasons for missing Week 12 data were primarily due to disease progression, adverse events, or death [9]. There were no differences in demographic or relevant clinical characteristics between those study patients with complete and missing Week 12 HRQL assessments. There were differences in baseline EORTC QLQ-C30 scores for global, physical, role, emotional, and social function scores and for fatigue, nausea, pain, dyspnea, sleep, appetite, and constipation scores between those with and without Week 12 assessments.

**Figure 1:**
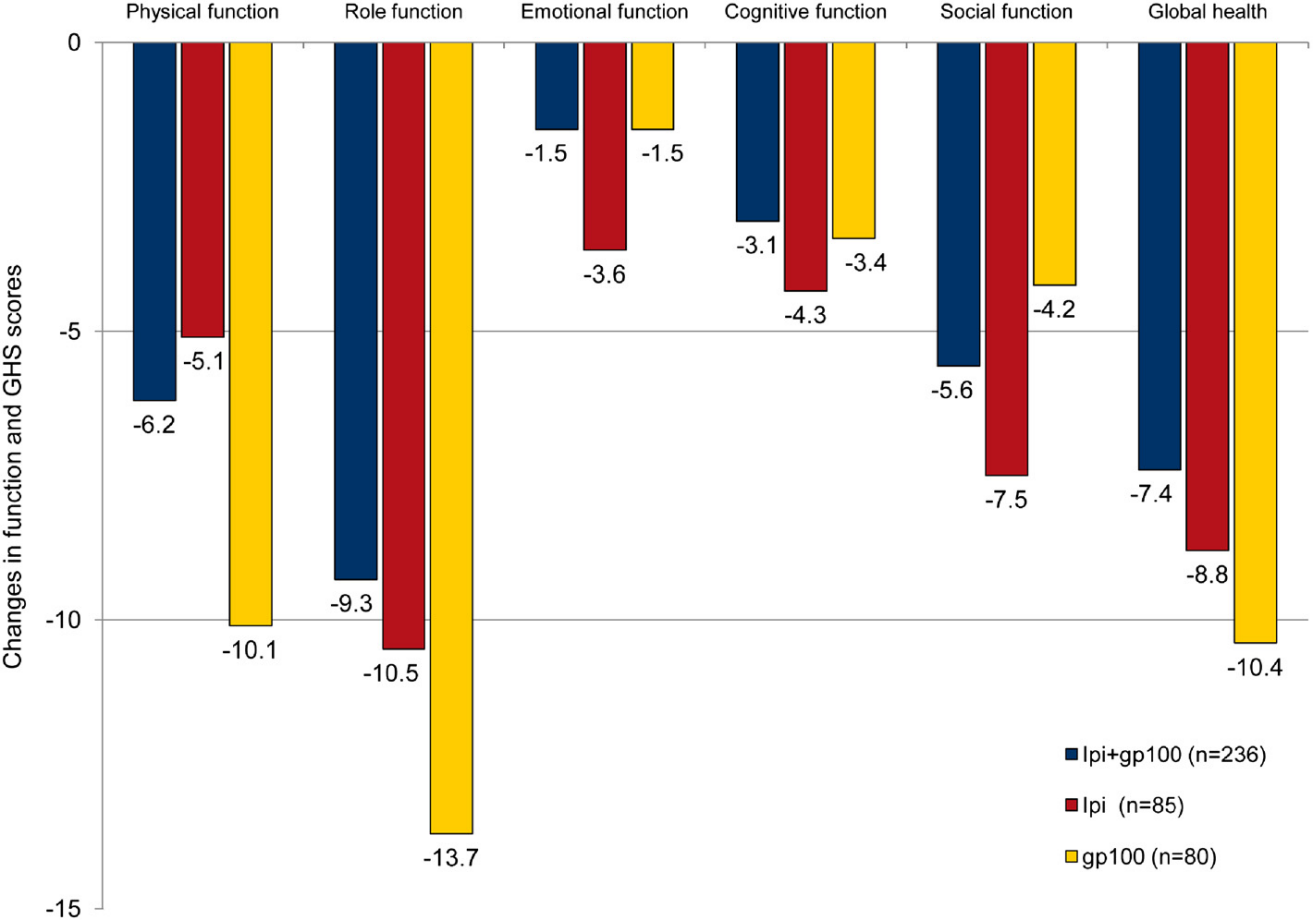
**Baseline to Week 12 endpoint changes in EORTC QLQ-C30 function and global health status scores.** * For the functioning and global health scales, improvements are indicated by positive scores. ** p > 0.05 for all comparisons. Mean change in scores were categorized as “no change” (0–5), “a little” (5–10 points), “moderate” (10–20 points), and “very much” (>20).

**Figure 2:**
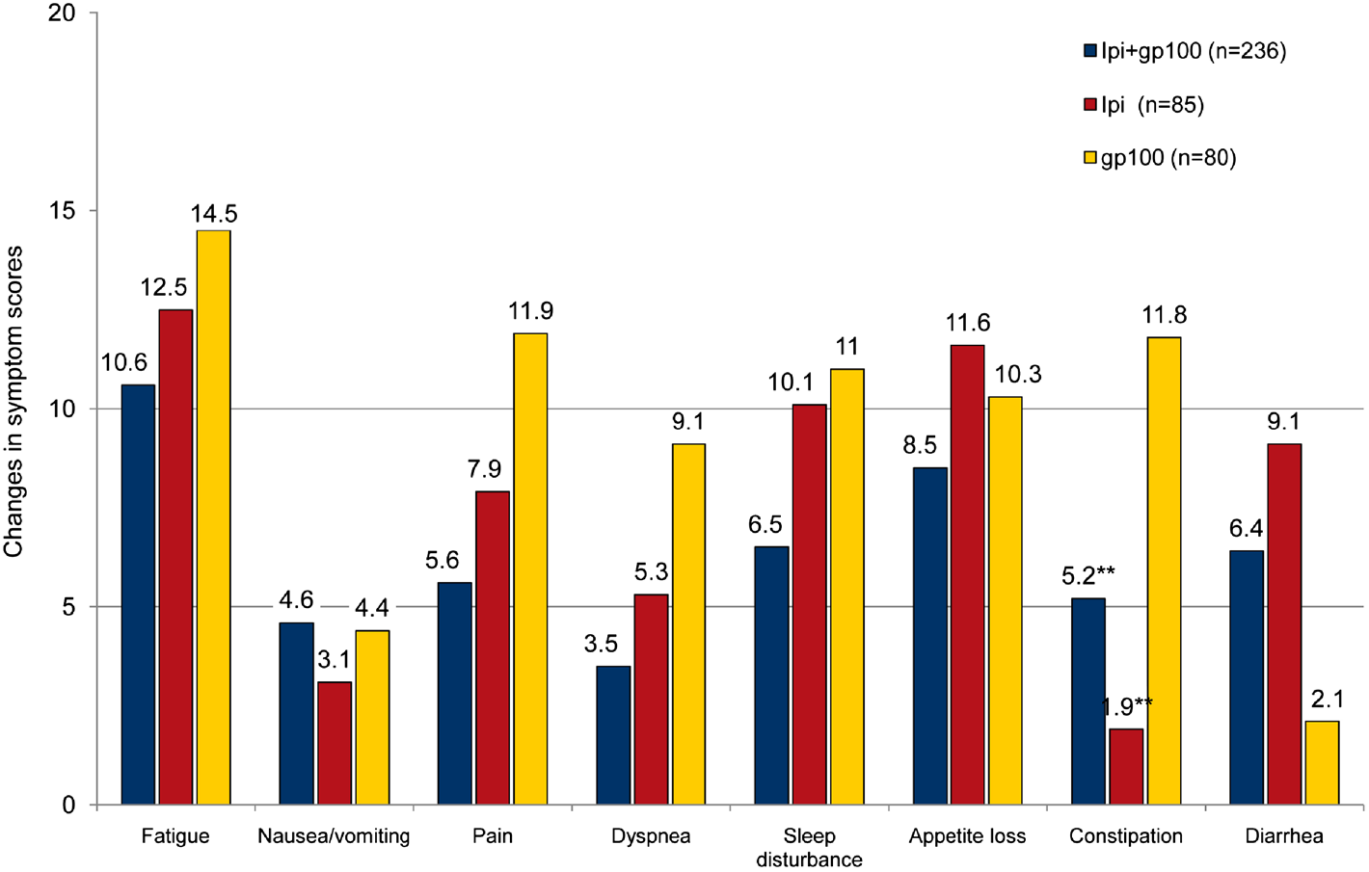
**Baseline to Week 12 endpoint changes in EORTC QLQ-C30 symptom scores.** * For symptom scales, improvements are indicated by negative scores. ** p < 0.05 versus gp100 group. *** p > 0.05 for all comparisons (except for Constipation with p < 0.05). Mean change in scores were categorized as “no change” (0–5), “a little” (5–10 points), “moderate” (10–20 points), and “very much” (>20).

When HRQL outcomes were evaluated, most observed baseline to Week 12 changes were no greater than minimal in the “a little” impairment category (Figures 1 and 2). There was a statistically significant difference in constipation scores between the ipilimumab plus gp100 and the gp100 alone groups (p < 0.05) and between the ipilimumab alone and gp100 alone groups (p < 0.05). None of the other differences in HRQL scores between the three treatments were statistically significant.

For the ipilimumab plus gp100 arm, the observed impairments were in the “no change” or “a little” categories for physical, role, emotional, cognitive, and social function, global health, nausea, pain, dyspnea, sleep disturbance, appetite loss, constipation, and diarrhea subscales. For the ipilimumab alone group, the observed impairments were in the “no change” or “a little” categories for the physical, emotional, cognitive, and social function, the global health, nausea, pain, dyspnea, constipation, and diarrhea subscales. For the gp100 alone group, the observed impairments were in the “no change” or “a little” categories for the cognitive and social function, nausea, dyspnea, and diarrhea subscales. In the gp100 alone group, moderate to large impairments were seen for global health, role function, fatigue, and pain.

Due to interest from clinicians in the analysis of results for older oncology patients, results from the post hoc subgroup analysis of EORTC QLQ-C30 data were also compared by two age groups: patients aged <65 years and those ≥65 years (Table 2). For patients <65 years, in the ipilimumab plus gp100 arm, there were none to small impairments in functional outcomes and symptom scores, while the older age group reported a similar pattern of changes for most of the outcomes. Older patients reported moderate impairments in role function, global health, fatigue and sleep disturbance. In the ipilimumab alone subgroup of patients <65 years, the impairment changes in functional outcomes and symptom scores were none to small for most scores, except for fatigue and appetite loss. For patients aged ≥65 years, in the ipilimumab alone group, moderate or greater impairments were seen in social function and global health, which differed somewhat from the younger age group. More symptom effects were observed in those ≥65 years for dyspnea and diarrhea compared with the <65 age group. These findings need to be interpreted cautiously given the smaller sample size.

**Table 2:** Baseline to week 12 endpoint changes in EORTC QLQ-C30 scores by age groups

	**Ipilimumab plus gp100**	**Ipilimumab plus gp100**	**Ipilimumab Alone**	**Ipilimumab Alone**
	**< 65 years**	**≥ 65 years**	**< 65 years**	**≥ 65 years**
	**(N = 170)**	**(N = 66)**	**(N = 59)**	**(N = 26)**
Physical function	−6.2	−9.5	−4.3	−9.3
Role function	−9.8	−11.7	−11	−12.9
Emotional function	−0.8	−6.2	−2.2	−9.3
Cognitive function	−3.9	−4.7	−3.6	−8.8
Social function	−5.4	−7.4	−6	−12.3
Global health	−6.5	−12.1	−6	−17
Fatigue	9	14.2	12.4	12.1
Nausea/vomiting	5.3	7.3	4.9	3.1
Pain	7.2	6.4	10	7.4
Dyspnea	2	8.1	2.1	12.5
Sleep disturbance	5	10.4	8.8	12.9
Appetite loss	9.4	9.6	12.9	11.7
Constipation	3.8	6	2.3	−0.4
Diarrhea	6.2	7.7	7.7	13.5

## Discussion

Ipilimumab at 3 mg/kg monotherapy, whether combined with gp100 vaccine or not, was associated with a 19% to 36% reduction in the rate of disease progression and, more importantly, had increased overall survival compared with the gp100 vaccine alone group in patients with previously treated advanced melanoma [9]. In general, the HRQL results for the ipilimumab groups demonstrate that ipilimumab treatment is associated with minimal impairments on functioning and symptoms during the treatment induction period. The only statistically significant difference between ipilimumab and gp100 vaccine was for constipation, and this finding may be due to increased rate of colitis in the ipilimumab groups (5.3-7.6% versus 0.8%) [9]. Most of the observed changes were in the range of “no change” or minimal impairments, which indicates that HRQL was maintained during the treatment induction period. Functioning and symptom scores did not improve during treatment; only the overall HRQL of these patients was negatively impacted to a small extent. The gp100 group reported increased pain, fatigue, dyspnea, and decreased physical and role function compared with the ipilimumab group.

After 12 weeks of treatment with ipilimumab, only fatigue, sleep disturbance, and appetite loss showed moderate impairments. However, there was no significant negative impact on physical, emotional, cognitive, and social functioning and global health status in the ipilimumab treated groups. These findings indicate that HRQL outcomes were minimally impaired by ipilimumab treatment. Therefore, the trade-offs between extended survival and HRQL may be acceptable to patients and their clinicians [45]. Given that few treatments for advanced melanoma (i.e., vemurafenib and ipilimumab) are associated with improvements in overall survival [9,10,12,13], these HRQL results for ipilimumab are very encouraging.

We identified three studies that used the EORTC QLQ-C30 comparing treatments for advanced melanoma [14,32,34]. Study design and methods are summarized in Additional file 1 Table A1. Two of these studies reported higher rates of missing HRQL data at follow-up compared with ipilimumab plus gp100 or ipilimumab alone (Additional file 1 Table A2). Disease progression rates were somewhat greater in the comparison studies, ranging from 61% to 74% (Additional file 1 Table A2). For the EORTC QLQ-C30 functional outcomes, dacarbazine-videsine-cisplatin and dacarbazine-videsine treated groups demonstrated worse global health and physical, role, and social function compared with ipilimumab plus gp100 or ipilimumab alone groups (Additional file 2 Figure A1). For the symptom outcomes, dacarbazine-videsine-cisplatin and dacarbazine-videsine treated groups demonstrated worse fatigue, nausea/vomiting, and appetite loss and similar pain compared with ipilimumab plus gp100 and ipilimumab alone groups (Additional file 3 Figure A2). The studies by Avril et al. [14] and Kiebert et al. [32] showed changes in EORTC QLQ-C30 function and symptom scores comparable to the ipilimumab plus gp100 and ipilimumab alone treatment groups. Overall, the ipilimumab HRQL effects we observed may be better or comparable to those observed in these other clinical trials, as supported by little meaningful impairment in functioning and symptoms during the treatment induction period.

The comparison of HRQL outcomes between the ipilimumab clinical trials and these other studies should be interpreted cautiously given the differences in methods, disease progression, and dropout rates. Significant differences in mechanism of action and known toxicity profiles of chemotherapy and ipilimumab may contribute to observed differences in HRQL between the chemotherapy and ipilimumab. In addition, ipilimumab’s demonstrated efficacy compared to the general lack of chemotherapy activity in this disease is another consideration for observed differences.

In the Phase II study for ipilimumab [38], mean changes from baseline to Week 12 for the 3 mg/kg arm generally indicated little or no negative impact to patient HRQL across the EORTC QLQ-C30 subscales for global health status, function, and symptoms. These Phase II results are similar to the Phase III (MDX010-20) results and add further support to the effects of ipilimumab treatment as possibly better or comparable to those observed in these other clinical trials.

Clinicians are concerned about the effects of treatment on elderly (i.e., ≥65 years of age) advanced melanoma patients [46,47]. Although overall survival is comparable for patients aged <65 and ≥65 years (for ipilimumab plus gp100 versus gp100 alone, hazard ratio was 0.70 and 0.69 for <65 years and ≥65 years, respectively; for ipilimumab alone versus gp100 alone, hazard ratio was 0.65 and 0.61 for <65 years and ≥65 years, respectively) [9], we evaluated differences in HRQL outcomes by age group. For the ipilimumab plus gp100, results were comparable for both age groups, although those ≥65 years reported more impairment in role function, global health, and sleep disturbance. For the ipilimumab alone groups, the results for functional outcome and symptom scores were comparable, except that those ≥65 years reported more impairment in social function, dyspnea, sleep disturbance, and diarrhea.

The HRQL results from the current ipilimumab study should be interpreted considering the following limitations. HRQL endpoint data were available for only 61% to 65% of patients randomized into the clinical trial. Disease progression was the most common reason for discontinuation of study drug (24% of subjects in the ipilimumab plus gp100 group; 16% in the ipilimumab alone group; and 33% in the gp100 alone group). Rates of discontinuation of study drug due to adverse events were greatest for ipilimumab alone (13%) compared with ipilimumab plus gp100 (9%) and gp100 alone (4%). However, there were comparable completion rates of the EORTC QLQ-C30 across treatment arms in the current study (MDX010-20), so these missing data may not impact the interpretation of the HRQL results. Missing HRQL data is a significant problem for oncology studies, and patients who complete follow-up assessments are less likely to experience severe toxicity and are more likely to have better response to treatment [37]. Finally, although EORTC QLQ-C30 is an internationally validated, widely used questionnaire for assessing the HRQL in oncology, melanoma specific HRQL questions might not have been addressed.

## Conclusions

In conclusion, ipilimumab at 3 mg/kg with and without gp100 vaccine does not have a significant negative impact on HRQL in patients completing the baseline and Week 12 follow-up, during the treatment induction phase compared with gp100 alone. Ipilimumab treatment results in little to no impairment in HRQL outcomes in advanced melanoma patients. The improved survival observed in the ipilimumab treated groups does not come with a significant burden on HRQL for patients in this analysis. Further research is needed to determine the long term impact of ipilimumab treatment on HRQL outcomes. In addition, further analyses are needed to better understand the impact of serious adverse events on HRQL in ipilimumab treated patients.

## Abbreviations

CNS, Central Nervous System; DTIC, Dacarbazine; ECOG, Eastern Cooperative Oncology Group; EORTC QLQ-C30, European Organization for Research and Treatment of Cancer Quality of Life Core Questionnaire; FACT, Functional Assessment of Cancer Therapy; HRQL, Health-Related Quality of Life; I.V., Intravenous.

## Competing interests

DAR and SS are employees of United BioSource Corporation and have research support from Bristol-Myers Squibb (BMS). AJMV is on the BMS advisory board and has been paid honoraria by BMS. PL is a consultant to BMS, has been a member of BMS Speakers Bureau, and has received honoraria from BMS to attend international conferences. PL has undertaken a number of unpaid academic projects with BMS around treatment of melanoma and also this study; which have been or are being submitted for publication. CL has received honoraria from BMS for ipilimumab development. GL is a consultant for GlaxoSmithKline, Genentech, and BMS and has received honoraria from BMS. CHO has consulted for BMS, received honoraria from BMS, and has received an unrestricted grant from BMS for a clinical trial using ipilimumab. MM declares that she has no competing interests. AH is an employee with BMS, holds a leadership position with the Cancer Immunotherapy Consortium, and owns stocks in BMS. SW is an employee with and owns stock in BMS. SK is an employee with, owns stock in, and has research funding from BMS.

## Authors' contributions

DAR participated in data analysis and interpretation and manuscript writing. AJMV participated in data analysis and interpretation and manuscript writing. PL participated in the collection and assembly of data, data analysis and interpretation, and manuscript writing. CL participated in the conception and design and manuscript writing. GL participated in data analysis and interpretation and manuscript writing. CHO participated in the conception and design and manuscript writing. SS participated in manuscript writing and provided project management and administrative support. MM participated in data analysis and interpretation and manuscript writing. AH participated in data analysis and interpretation and manuscript writing. SW participated in conception and design and manuscript writing. SK participated in the conception and design, collection and assembly of data and manuscript writing. All authors read and approved the final manuscript.

## Supplementary Material

Additional file 1 **Methodology for comparison with other melanoma clinical trials.** A systematic search of review articles and clinical trial articles was conducted in order to compare the HRQL findings from the ipilimumab studies to other published clinical trials in advanced melanoma that included the EORTC QLQ-C30. Microsoft Word document file name: HRQL Melanoma Manuscript Appendix_HQLO_final.doc. Click here for file

Additional file 2 **Figure A1.** Baseline to endpoint changes in EORTC QLQ-C30 function and global health scores for advanced melanoma studies. Click here for file

Additional file 3 **Figure A2.** Baseline to endpoint changes in EORTC QLQ-C30 symptom scores for advanced melanoma studies. Click here for file
