# Supplementary material for: Health related quality of life outcomes for unresectable stage III or IV melanoma patients receiving ipilimumab treatment
Source: Health Qual Life Outcomes. 2012 Jun 13;10:66. doi: 10.1186/1477-7525-10-66 (PMC3426458; doi:10.1186/1477-7525-10-66)
Supplement: Additional file 1 — Methodology for comparison with other melanoma clinical trials. A systematic search of review articles and clinical trial articles was conducted in order to compare the HRQL findings from the ipilimumab studies to other published clinical trials in advanced melanoma that included the EORTC QLQ-C30. Microsoft Word document file name: HRQL Melanoma Manuscript Appendix_HQLO_final.doc. [file 1477-7525-10-66-S1.doc]

**Methodology for comparison with other melanoma clinical trials**

A systematic search of review articles and clinical trial articles in peer-reviewed journals was conducted to identify clinical trials in advanced melanoma which assessed HRQL using the EORTC QLQ-C30 or other HRQL instruments. This review initially identified 477 potential articles fitting the review criteria. Careful review of the article abstracts yielded 11 publications that included either the EORTC QLQ-C30 or Functional Assessment of Cancer Treatment (FACT) measures as assessments of HRQL outcomes. Three of these studies were advanced melanoma clinical trials that included the EORTC QLQ-C30. We compared the HRQL findings from the ipilimumab studies to these other published clinical trials in advanced melanoma that included the EORTC QLQ-C30.

***Table A1. Brief protocol descriptions for other treatments for advanced melanoma***

| **Sigurdardottir et al. (1996)** | **Kiebert et al. (2003)** | **Avril et al. (2004)** |
| --- | --- | --- |
| **Objective:** To compare HRQL outcomes during chemotherapy in patients with advanced melanoma.  **Treatment:** Patients were randomized to receive either dacarbazine 250 mg/m^2^ intravenous (i.v.) Days 1-5 every 4 weeks and vindesine 3mg/m^2^ i.v. Days 1, 8, 15 and 22, with or without cisplatin 100mg/m^2^ i.v. Day 1 every 4 weeks.  **HRQL Assessment:** The EORTC QLQ-C36 (the interim version of the questionnaire) was administered at baseline, on Day 15 of the second course, on Day 8 of the third course (Week 9), on Day 1 of the fourth course (Week 12), and on Day 1 of every second course thereafter until the treatment was discontinued. The EORTC QLQ-C30 change scores for treatment induction period were evaluated at  Week 9. | **Objective:** To compare HRQL outcomes between temozolomide and Dacarbazine (DTIC) in patients with metastatic melanoma.  **Treatment:** Patients were randomized to receive either oral temozolomide for 5 days every 4 weeks or i.v. DTIC for 5 days every 3 weeks.  **HRQL Assessment:** The EORTC QLQ-C30 (version not specified) was measured on Day 1 of cycle 1 and after completion of each subsequent cycle. The EORTC QLQ-C30 change scores for the treatment induction period were evaluated at Week 12. | **Objective:** To compare the HRQL outcomes between fotemustine and DTIC in patients with disseminated cutaneous melanoma.  **Treatment:** Patients were randomized to receive via i.v. either fotemustine or DTIC. Fotemustine was administered weekly for 3 consecutive weeks (Days 1, 8, and 15) at 100 mg/m^2^ as a 60-minute infusion, followed by a 5-week rest period (defined as induction treatment). DTIC was administered as a 60-minute infusion once a day for 5 consecutive days at 250 mg/m^2^ every 4 weeks (Days 1 to 5 and 29 to 33), for two cycles as induction treatment.  **HRQL Assessment:** The EORTC QLQ-C30 (version 2.0) was administered before random assignment in both arms, at the end of the induction cycle, at each maintenance cycle, then at the end of the study in the fotemustine arm and at each cycle and end of study in the DTIC arm. The EORTC QLQ-C30 change scores for the treatment induction period were evaluated at Week 7 for Fotemustine and Week 8 for DTIC. |

***Table A2. Disease progression and dropout rates for ipilimumab and other treatments for advanced melanoma***

| **Study** | **Treatment Group** | **Dropout Rates (%)** | **Progressive Disease (%)** |
| --- | --- | --- | --- |
| Phase III (MDX010-20) | Ipilimumab plus gp100  Ipilimumab alone | 38  35 | 59  51 |
| Sigurdardottir et al. (1996) | Randomized to clinical trial  Dacarbazine-vindesine-cisplatin  Dacarbazine-vindesine | 54  42 | 41% (at Week 9) |
| Kiebert et al. (2003)* | Temozolomide  DTIC | 68  79 | 61  63 |
| Avril et al. (2004) | Fotemustine  DTIC | 26  38 | 66  74 |

* Disease progression data is reported in Middleton et al. study (original clinical trial publication)

## Appendix figures

## Figure A1. Baseline to endpoint changes in EORTC QLQ-C30 function and global health scores for advanced melanoma studies

* For the functioning and global health scales, improvements are indicated by positive scores

** Please note that the following subscales were not reported for Figure A1: Cognitive Function data from both Sigurdardottir et al. (1996) studies; Emotional & Cognitive Function data from Avril et al. (2004) study.

*** Change scores for Kiebert et al. (2003) were not provided in the article for all domains, therefore, change scores were calculated by using the values provided in Table 3 of Kiebert article (i.e., subtract baseline mean value from Week 12 mean value).

**** Kiebert et al. (2003) reports baseline to Week 12 endpoint changes; Sigurdardottir et al. (1996) reports baseline to Week 9; Avril et al. (2004) reports baseline to Week 7 (Fotemustine) or Week 8 (Dacarbazine [DTIC]).

Mean change in scores were categorized as “no change” (0-5), “a little” (5-10 points), “moderate” (10-20 points) and “very much” (>20)

## Figure A2. Baseline to endpoint changes in EORTC QLQ-C30 symptom scores for advanced melanoma studies

* For symptom scales, improvements are indicated by negative scores

** Please note that the following subscales were not reported for Figure A2: Dyspnea, Sleep Disturbance, Constipation, and Diarrhea data from both Sigurdardottir et al. (1996) studies; Dyspnea, Sleep Disturbance, Appetite Loss, Constipation, and Diarrhea data from Avril et al. (2004) study.

*** Change scores for Kiebert et al. (2003) were not provided in the article for all domains, therefore, change scores were calculated by using the values provided in Table 3 of Kiebert article (i.e., subtract baseline mean value from Week 12 mean value).

**** Kiebert et al. (2003) reports baseline to Week 12 endpoint changes; Sigurdardottir et al. (1996) reports baseline to Week 9; Avril et al. (2004) reports baseline to Week 7 (Fotemustine) or Week 8 (DTIC).

Mean change in scores were categorized as “no change” (0-5), “a little” (5-10 points), “moderate” (10-20 points) and “very much” (>20)

**Figure A3. Consort Diagram**

Included in primary analysis (n=137)

Evaluated for adverse events (n=131)

Evaluated for response (n=109)

Missing tumor assessments (n=22)

Deaths (n=100)

Included in primary analysis (n=403)

Evaluated for adverse events (n=380)

Evaluated for response (n=320)

Missing tumor assessments (n=61)

Deaths (n=306)

Included in primary analysis (n=136)

Evaluated for adverse events (n=132)

Evaluated for response (n=104)

Missing tumor assessments (n=27)

Deaths (n=119)

Received at least one treatment (n=131)
Received all scheduled treatments (n=88)

Discontinued study drug (n=43)

Adverse events (n=17)

Disease progression (n=21)

Other (n=5)

Received at least one treatment (n=380)

Received all scheduled treatments (n=245)

Discontinued study drug (n=135)

Adverse events (n=34)

Disease progression (n=93)

Other (n=8)

Received at least one treatment (n=132)^‡^

Received all scheduled treatments (n=78)

Discontinued study drug (n=54)

Adverse events (n=5)

Disease progression (n=43)

Other (n=6)

Did not start treatment (n=6)

Died (n=6)

Did not start treatment (n=22)

Died (n=17)

Withdrew consent (n=4)

Decline in performance status (n=1)

Received only gp100^†^ (n=1)

Did not start treatment (n=5)

Died (n=2)

Protocol violation (n=1)

Decline in performance status (n=1)

Investigator’s decision (n=1)

Assigned to gp100 alone (n=136)

Assigned to ipilimumab alone (n=137)

Assigned to ipilimumab plus gp100 (n=403)

HLA-A*0201-positive patients with unresectable stage III or stage IV melanoma enrolled (n=676)

^†^This patient was included in the gp100 alone group for safety analyses. ^‡^Includes 1 patient randomized to ipilimumab plus gp100 who received only gp100.
