# Supplementary figures and images for: Health related quality of life outcomes for unresectable stage III or IV melanoma patients receiving ipilimumab treatment
Source: Health Qual Life Outcomes. 2012 Jun 13;10:66. doi: 10.1186/1477-7525-10-66 (PMC3426458; doi:10.1186/1477-7525-10-66)

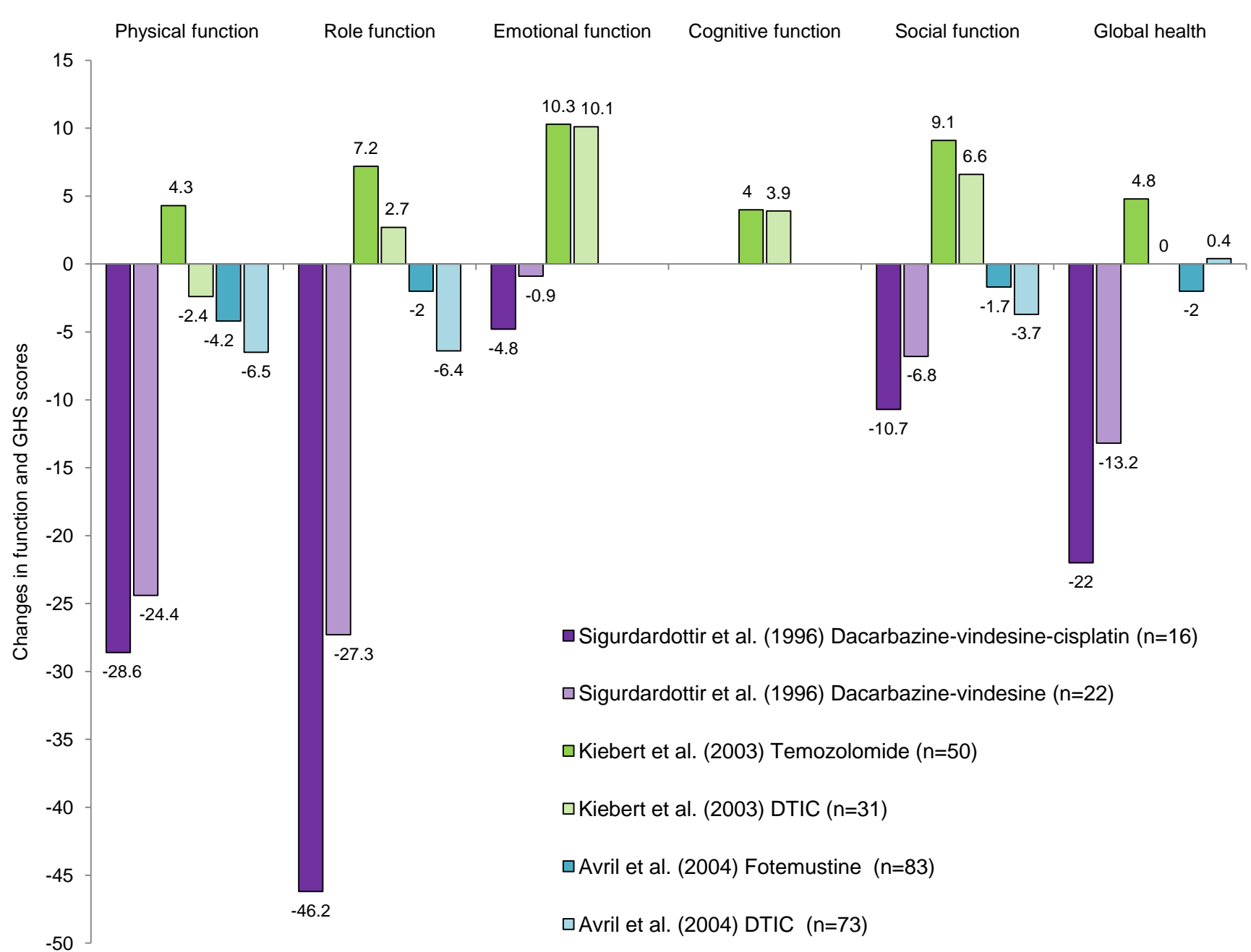

Supplement: Additional file 2 — Figure A1. Baseline to endpoint changes in EORTC QLQ-C30 function and global health scores for advanced melanoma studies. [file 1477-7525-10-66-S2.pdf]

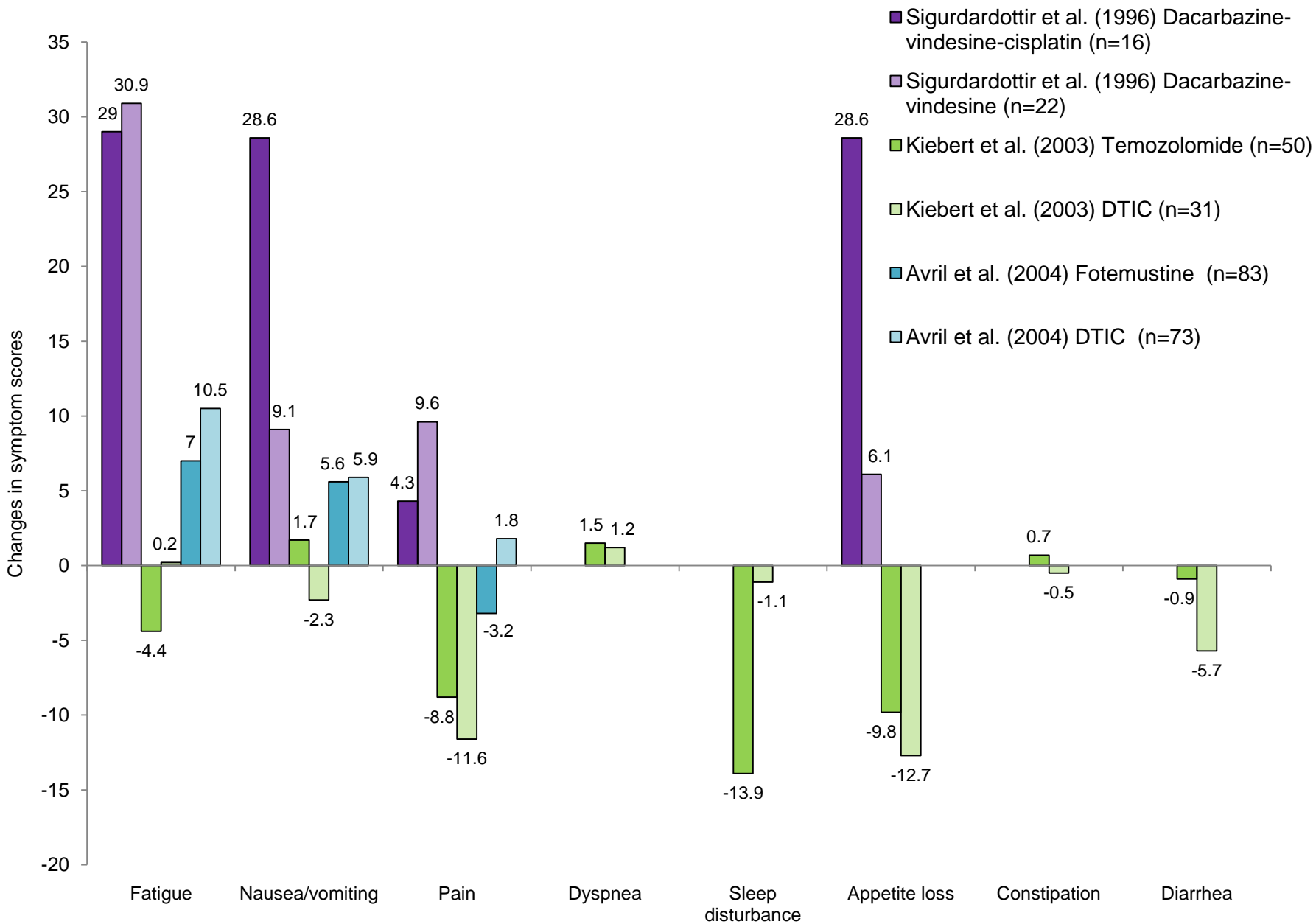

Supplement: Additional file 3 — Figure A2. Baseline to endpoint changes in EORTC QLQ-C30 symptom scores for advanced melanoma studies. [file 1477-7525-10-66-S3.pdf]
